# Migration from Latin America to Italy and EU: the issue of health professionals: Elisabetta Corvo

**DOI:** 10.1093/eurpub/ckac131.380

**Published:** 2022-10-25

**Authors:** W De Caro, E Corvo

**Affiliations:** 1 CNAI Italian Nursing Association, Rome, Italy; 2 Canterbury Christ Church, Canterbury, UK

## Abstract

**Introduction:**

The estimated shortage of more than 15 million health and social care professionals, including over 6 million nurses, together with the significant phenomenon of international migration of such professionals, emerged, during COVID-19, with even more evidence. During the pandemic the danger of a collapse of health systems in many areas due to a shortage of staff was manifest. This leads, among other reasons for several professionals to migrate from South America to Italy and Europe.

**Methods:**

an integrative review analysis (2011-2021), of trend in terms of legislation and policy documents were conducted using health and social database and gray literature. 34 different documents were included in this review.

**Results:**

Several initiatives have been developed to establish international and/or bilateral obligations among countries in terms; however, this showed to be insufficient. There is confusion in terms of the necessity of harmonizing rules, nursing curricula, monitor systems, and innovative rules for recognition. With covid-19 and the increase in shortage of health professionals, a number of countries decide to reduce the general criteria of recognition.

**Conclusions:**

It is time to develop specific actions aimed at setting up a passport for public health and health care professionals which allows guaranteeing prior certification and accreditation of skills by independent agencies. There is the need to set up a joint effort/actions among different stakeholders such as Government, Higher Education institutions, International Labor health market actors, and NGOs in the field of migration, labor, and training of public health and health care professionals to better meet health needs worldwide

**Key messages:**

• it is necessary balancing the rights of individuals to migrate with the potential loss of essential health care services in source countries.

• migration and shortage of nurses is a serious ethical issues.

